# PReS-FINAL-2091: Extra-articular calcification after intra-articular corticosteroid injection

**DOI:** 10.1186/1546-0096-11-S2-P103

**Published:** 2013-12-05

**Authors:** P Toftedal, LA Bistrup, AE Christensen

**Affiliations:** 1 Hans Christian Andersen Children's Hospital, Odense University Hospital, Odense, Denmark

## Introduction

Intra-articular corticosteroid injection is a well established therapeutic option for treatment of juvenile idiopathic arthritis (JIA). Subcutaneous atrophy and depigmentation are well recognised adverse effects. Peri-articular calcification has been reported in radiological studies and the majority was asymptomatic.

## Objectives

Two cases with pain and thickness around joints due to extra-articular calcification are reported in order to increase the awareness of this side effect.

## Methods

Case report.

## Results

Two adolescent girls with polyarticular JIA reported pain and swelling around the metacarpophalangeal (MCP) and interphalangeal (IP) joints without symptoms of arthritis in other joints. Both girls were treated with methotrexate and anti-TNF-alpha agents for years. Several flares of arthritis had been seen in both small and large joints. No extra-articular symptoms were reported. Both patients had been treated by several intra-articular corticosteroids injections with triamcinolone hexacetonide including the MCP and IP joints. No adverse effects had been noticed.

X-ray of the hands showed periarticular or capsular calcification of some MCP and IP joints in both patients. MRI showed no signs of arthritis and no damage on intra-articular structures.

## Conclusion

Intra-articular injection in JIA is a safe and rapidly effective treatment for synovitis. Extra-articular calcification, clinically mimicking arthritis, is reported as an adverse event to corticosteroid injection. Risk of extra-articular side effects is probably higher in small joints.

## Disclosure of interest

None declared.

